# The prevalence of self-injury in adolescence: a systematic review and meta-analysis

**DOI:** 10.1192/j.eurpsy.2024.404

**Published:** 2024-08-27

**Authors:** B. F. Farkas, Z. K. Takács, N. Kollárovics, J. Balázs

**Affiliations:** ^1^Doctoral School, Semmelweis University; ^2^Institute of Education, Eötvös Loránd University, Faculty of Education and Psychology, Budapest, Hungary; ^3^School of Health in Social Sciences, University of Edinburgh, Edinburgh, United Kingdom; ^4^Institute of Psychology, Department of Developmental and Clinical Child Psychology, Eötvös Loránd University, Faculty of Education and Psychology, Budapest, Hungary; ^5^Oslo New University College, Oslo, Norway

## Abstract

**Introduction:**

Self-injurious behavior (SIB) among adolescents has become a hot topic in psychiatry. Despite the consensus that the prevalence of SIB is high, 26-22% among adolescence, there are conflicting results about whether it has increased in the 21st century and about the global distribution of the prevalence.

**Objectives:**

The aim of the current study was to make a systematic search and meta-analysis of publications from the last 5 years on the prevalence of SIB in adolescents and to examine definitions and assessments of SIB, gender, continental, and year differences. The hypotheses were the following: 1) the prevalence of SIB did not change over time between the examined period for both girls and boys; 2) girls reported a higher prevalence of a history of SIB than boys.

**Methods:**

The systematic search was made in June 2020. Six databases were used. The main search terms were “self-injurious behavior”, “prevalence” and “adolescence”. First the titles and abstracts of the relevant articles were checked, then the full texts were read and collected those papers that met the inclusion criteria. The inclusion criteria were the following: published between 01/01/2015, and 06/18/2020, focused on community sample, and written in English. Comprehensive Meta-Analysis software was used to conduct the analyses.

**Results:**

In sum, a total of 97 articles were included in the meta-analysis with data from 439 818 participants. The overall average SIB prevalence was 16.0% in these studies. The first hypothesis was only partially confirmed. When all data that were published between 2015 and 2018 were considered, x significant increase was found in the prevalence of SIB between 1998 and 2018. However, when the analysis was restricted to the time frame between 2013 and 2018, no change in prevalence was found. The second hypothesis was fulfilled, girls reported a significant higher prevalence than boys (19.4% and 12.9%, respectively). A significantly higher prevalence was found when suicidal intent was excluded (18.3%), than when it was not excluded (11.3%) from the definition of SIB. The largest prevalence was found when measurement instruments were used that had been validated for SIB (18.9%). A significantly higher SEB prevalence was found among Asian articles than those from other continents (19.5% and 14.7% respectively).

**Conclusions:**

The current systematic review and meta-analysis draw attention to the high prevalence of SIB among adolescents, especially among girls and those living in Asia. It is important to address this behavior, in terms of prevention and intervention as well.

**Disclosure of Interest:**

None Declared

